# Draft genome sequence of multidrug-resistant *Klebsiella pneumoniae* Hakim-RU strain isolated from a patient with urinary tract infections in Bangladesh

**DOI:** 10.1128/mra.00089-24

**Published:** 2024-03-27

**Authors:** M. Romance, Muhib Ullah khan, Md. Shamsul Islam, Md. Faridul Islam, Md. Hakimul Haque

**Affiliations:** 1 Department of Veterinary and Animal Sciences, University of Rajshahi, Rajshahi, Bangladesh; Loyola University Chicago, Chicago, Illinois, USA

**Keywords:** whole genome, urinary tract infections, multidrug resistant, *Klebsiella pneumoniae*, Bangladesh

## Abstract

We unveil the genomic sequence of the *Klebsiella pneumoniae* Hakim-RU strain isolated from a patient with urinary tract infections. Our assembled genome spans 4.3 Mb with 73.0× coverage, an average GC content of 57.41%, 4 plasmids, 2 CRISPR arrays, 10 prophages, 41 antibiotic resistance genes, and 6 virulence factor genes.

## ANNOUNCEMENT

*Klebsiella pneumoniae* ranks the second-most prevalent opportunistic pathogen, causing approximately 150 million urinary tract infections (UTIs) annually (1, 2). UTIs posed by this bacterium are exacerbated by multidrug resistance resulting from the improper use of prescription drugs and substandard pharmaceuticals (3). As one of the ESKAPE pathogens, *K. pneumoniae* presents a substantial threat in causing life-threatening nosocomial infections among critically ill and immunodeficient patients (4). Previously, antimicrobial-resistant *K. pneumoniae* was isolated from humans, animals, foods, and environments throughout the globe (5–8). Therefore, the escalating rates of antimicrobial resistance globally have led to increased surveillance and molecular epidemiological research focusing on *K. pneumoniae* in UTI patients (9, 10).

All research techniques and protocols were ethically approved by the Institute of Biological Science (IBSc) at the University of Rajshahi, Bangladesh (Memo No. 56/321/IAMEBBC/IBSc). Between November 2021 and December 2022, midstream urine samples were collected from UTI patients in the Rangpur District of Bangladesh (25.7468°N, 89.2508°E) following standard procedures. The urine samples were uniformly mixed, placed in sterile glass jars, and transported to the laboratory (24.3733°N, 88.6049°E). These urine samples were inoculated on a UTI agar (HiMedia, India) and aerobically incubated at 37°C for 18–24 hours (11). Isolation of *K. pneumoniae* was done by streaking the cultures on MacConkey (HiMedia, India) and Eosin Methylene Blue agar (HiMedia, India), followed by staining and biochemical tests (12)). The antimicrobial susceptibility of the isolates was determined using the disk diffusion method (13) and following the CLSI guidelines (14). The strain displays phenotypic resistance to amoxicillin**,** amoxicillin + clavulanic acid, ceftriaxone, cephradine, cefuroxime sodium, cefixime, co-trimoxazole, azithromycin, ciprofloxacin, and nalidixic acid. The isolate was cultured in nutrient broth (HiMedia, India) at 37°C overnight, and its genomic DNA was extracted using the Qiagen DNA Mini Kit (QIAGEN, Hilden, Germany). Genomic DNA underwent enzymatic fragmentation using the NEBNext dsDNA Fragmentase Kit (NEB, MA, USA), followed by size selection with SPRI beads (15). A sequencing library was generated using the Nextera DNA Flex Library Prep Kit (Illumina, San Diego, CA, USA), and the library was sequenced with 2 × 150 paired-end reads on the Illumina NextSeq2000 platform. Genome assembly was performed using Unicycler.v0.4.9 (16), and raw paired-end reads (*n* = 9,226,572) were trimmed with Trimmomatic.v0.39 (17). Quality checks were conducted using FastQC v0.11.7 (18), and annotation was performed using PGAP v3.0 (19). The assembled genome was analyzed for antibiotic resistance genes (ARGs) using CARD v.3.2.4 with RGI v6.0.2 (20) and ResFinder v.4.1 (21), plasmids using PlasmidFinder v.2.1 (22), virulence factor genes using VFDB with VFanalyzer (23), pathogenicity index using PathogenFinder v.1.1 (24), sequence type using MLST v.2.0 (25), CRISPR arrays using CRISPRimmunity (26), prophages using PHASTER (27), and metabolic functional features using RAST v.2.0 (28). Default parameters were employed for all tools unless otherwise specified.

**Fig 1:**
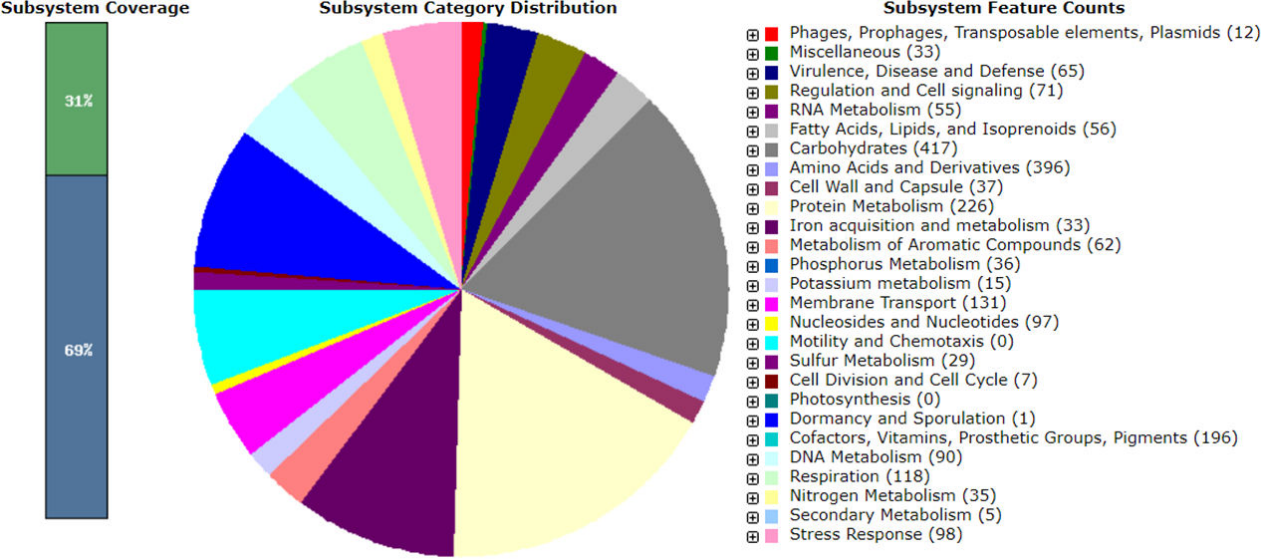
Metabolic functional features in the assembled genome of the *K. pneumoniae* Hakim-RU strain.

The features of the draft genomes are noted in Table 1. Notably, 41 ARGs, 6 virulence genes, and 4 plasmids [ColpVC, IncFIB(K), IncFII(K), repB(R1701)] were identified. MLST classified the genome as sequence type 1 (ST1), and the PathogenFinder tool indicated a pathogenicity index of 0.891. The genome exhibited 2 CRISPR arrays with signature genes (*DEDDh, cas3*) and 10 prophages. RAST analysis uncovered 393 subsystems, comprising 5,289 genes with 31% coverage (Fig. 1).

**TABLE 1:** Genomic features of the *K. pneumoniae* strain Hakim-RU

Elements	Values
Genome size	5,361,640 bp
Genome coverage	73.0×
G + C content	57.41%
Number of contigs	92
Contig L50	5
Contig N50	371,239 bp
Total genes	5,303
Coding sequences	5,214
Coding genes	5,096
RNA genes	89
tRNA genes	77
rRNA genes	2
mtRNA genes	1
ncRNA genes	9
Pseudo genes	118
Genes assigned to SEED subsystems	5,289
Number of subsystems	393

## Data Availability

The study on *Klebsiella pneumoniae* Hakim-RU, conducted using the WGS shotgun approach, was submitted to NCBI/GenBank, and it was assigned the accession number JAZBEW000000000. The pertinent data, including the original readings, were stored with BioProject accession number PRJNA1040244, BioSample accession number SAMN38250261, and SRA accession number SRR27586253. The specific version mentioned in this document is labeled as JAZBEW000000000.1.
